# Artificial Intelligence for Tuberculosis Screening and Detection: From Evidence to Policy and Implementation

**DOI:** 10.3390/diagnostics16081127

**Published:** 2026-04-09

**Authors:** Hien Thi Thu Nguyen, Vang Le-Quy, Anh Tuan Dinh-Xuan, Linh Nhat Nguyen

**Affiliations:** 1 Department of Molecular Diagnostics, Aalborg University Hospital, 9260 Aalborg, Denmark; 2 Department of Clinical Medicine, Aalborg University, 9260 Aalborg, Denmark; 3 AVSE Global Medical Translational Research Network, 75008 Paris, France; 4 Novodan ApS, 9220 Aalborg, Denmark; 5 AVSE Global Data Science Network, 75008 Paris, France; 6 Department of Respiratory Medicine and Physiology, Cochin University Hospital, 75005 Paris, France; anh-tuan.dinh-xuan@aphp.fr; 7 Department for HIV, Tuberculosis, Hepatitis and Sexually Transmitted Infections, World Health Organization (WHO) Headquarters in Geneva, 1211 Geneva, Switzerland

**Keywords:** tuberculosis, artificial intelligence, computer-aided detection (CAD), chest radiography, screening and triage

## Abstract

Artificial intelligence (AI) is increasingly used to support tuberculosis (TB) screening and diagnosis, particularly through computer-aided detection (CAD) applied to chest radiography (CXR). However, the programmatic value of AI depends not only on diagnostic accuracy but also on implementation context, threshold calibration, and integration into diagnostic pathways. We conducted a narrative, state-of-the-art review of AI applications across the TB diagnosis pathway. Evidence was synthesized from World Health Organization policy documents, independent validation initiatives, and peer-reviewed studies published between 2010 and 2026, with a structured selection process aligned with PRISMA principles. CAD for CXR is the most mature AI application and is recommended by WHO for TB screening and triage among individuals aged ≥15 years in specific contexts. Across studies, CAD-CXR demonstrates sensitivity comparable to human readers, although performance varies by product, population, and imaging conditions, necessitating local threshold calibration. Evidence from implementation studies suggests improvements in screening efficiency and potential cost-effectiveness in high-burden settings. Other AI modalities, including computed tomography (CT)-based imaging analysis, point-of-care ultrasound interpretation, cough or stethoscope sound analysis, clinical risk models, and genomic resistance prediction show promising but heterogeneous results, with most requiring further independent validation and prospective evaluation. AI has the potential to strengthen TB screening and diagnostic pathways, but its impact depends on integration into health systems and evaluated using patient- and program-level outcomes rather than accuracy alone. A differentiated approach is needed, with responsible scale-up of policy-endorsed tools alongside rigorous evaluation of emerging technologies to support effective and equitable TB care.

## 1. Introduction

Tuberculosis (TB) continues to impose a substantial global health and economic burden despite the availability of effective diagnostics and treatment. Persistent gaps in case detection, together with delays from symptom onset to treatment initiation, continue to limit the impact of national TB programs [1,2]. These challenges are further compounded by structural constraints, including shortages of trained readers, inconsistent interpretation of chest radiographs (CXR), limited capacity for confirmatory testing, and logistical barriers that disproportionately affect high-risk and underserved populations [2].

In this context, artificial intelligence (AI) has emerged as a pragmatic tool to support TB screening and diagnosis rather than to replace clinical judgment. Current AI applications aim to standardize image interpretation, improve early sensitivity within the diagnostic cascade, and reduce delays between screening and confirmation testing [3]. The most advanced and widely implemented application to date is computer-aided detection (CAD) for digital CXR, which is used for TB screening and triage by identifying individuals who should undergo confirmatory microbiological testing [4].

When appropriately calibrated and integrated into diagnostic workflows, CAD-CXR may reduce reader workload, and support more standardized triage in high-throughput screening settings, which has contributed to its policy endorsement for adult TB screening in selected contexts [5,6]. However, improvements in diagnostic performance alone are insufficient to ensure patients benefit unless AI tools are embedded within well-functioning diagnostic and treatment pathways [7].

Beyond CXR-based screening, a range of AI-enabled approaches is under investigation across the TB diagnostic pathway, including computed tomography, point-of-care ultrasound, acoustic analysis of cough or lung sounds, clinical risk stratification models, and genomic resistance prediction [8,9,10,11,12]. However, these tools differ substantially in their levels of evidence, readiness for routine use, and relevance to programmatic decision-making, and most remain at the research or pilot stage [1].

This article presents a narrative, state-of-the-art review of AI applications across the TB diagnosis pathway, spanning screening, triage, diagnosis, and drug-resistance prediction. We synthesize evidence from international policy and guidance, independent validation initiatives, and a broad body of peer-reviewed studies reporting diagnostic performance, implementation experience, and patient- and program-level outcomes [4,13]. Particular emphasis is placed on CAD-enabled chest radiography, given its current policy endorsement and increasing programmatic adoption [1], while other AI modalities, including CT imaging, ultrasound, acoustic analysis, risk stratification models, and genomic tools, are critically appraised in relation to their evidence maturity, generalizability, and readiness for real-world deployment. By integrating technical performance with implementation, economic, and health system considerations, this review aims to provide a programmatically relevant framework for evaluating the role of AI in tuberculosis control.

## 2. Materials and Methods

This article presents a narrative and state-of-the-art review of AI applications for TB screening and diagnosis. The objective was to synthesize current evidence from policy documents, independent validation initiatives, and peer-reviewed research studies to provide a programmatically oriented overview of AI technologies across the TB diagnostic pathway. Although the review was not designed as a formal systematic review or meta-analysis, the identification and selection of evidence followed a structured and transparent approach aligned with key principles of the Preferred Reporting Items for Systematic Reviews and Meta-Analyses (PRISMA) framework to enhance reproducibility and reduce selection bias [14].

### 2.1. Information Sources and Search Strategy

Evidence was identified from multiple complementary sources to capture both policy guidance and peer-reviewed scientific literature. These included:

World Health Organization (WHO) policy documents and technical guidance related to TB screening, diagnosis, and AI-enabled software as a medical device [3].

Outputs from independent validation initiatives, including benchmarking platforms evaluating computer-aided detection (CAD) software systems for chest radiography.

Bibliographic databases including PubMed/MEDLINE and Google Scholar.

Bibliographic searches covered the period January 2010 to January 2026, reflecting the rapid evolution of deep learning methods applied to medical imaging during the past decade. Search terms were combined using Boolean operators and included variations in the following keywords: “tuberculosis”, “computer-aided detection”, “chest radiography”, “artificial intelligence”, “deep learning”, “screening”, “triage”, “cost-effectiveness”, and “implementation”.

Reference lists of key publications and relevant review articles were also screened to identify additional studies not captured through database searches. Gray literature was considered when it originated from major international organizations involved in TB policy or diagnostic evaluation.

### 2.2. Eligibility Criteria

Studies were considered eligible for inclusion if they met the following criteria:

- Investigated artificial intelligence or machine learning approaches applied to TB screening, diagnosis, risk prediction, or drug-resistance detection;
- Reported diagnostic accuracy, implementation outcomes, operational feasibility, economic evaluation, or program-level impact;
- Were published in peer-reviewed journals or authoritative technical reports from recognized global health institutions;
- Were available in English.

Studies were excluded if they:

- Focused exclusively on non-tuberculosis respiratory diseases without TB-specific analysis,
- Lacked sufficient methodological detail to interpret results,
- Consisted solely of opinion pieces or editorials without original analysis or structured synthesis.

Because the objective of this review was to inform policy and programmatic implementation rather than conduct pooled statistical analyses, studies were not excluded based solely on study design. Both experimental and observational studies were considered.

### 2.3. Study Screening and Selection

A total of 594 records were identified through database searching and additional sources (PubMed/MEDLINE, *n* = 274; Google Scholar, *n* = 300 [most relevant results]; other sources, *n* = 20). After removal of 156 duplicate records, 438 articles were screened based on title and abstract.

Of these, 319 records were excluded because they were not relevant to tuberculosis, did not involve AI-based approaches applicable to screening or diagnosis, or lacked clinical or programmatic relevance.

A total of 119 full-text articles were assessed for eligibility. Following full-text review, 53 articles were excluded due to insufficient methodological detail, lack of relevant outcomes, or non-TB-specific focus.

In total, 66 studies were included in the final qualitative synthesis.

Screening and selection were conducted independently by two reviewers to improve consistency in study identification. Disagreements regarding study inclusion were resolved through discussion and consensus.

### 2.4. Data Extraction and Synthesis

Data from included sources were extracted narratively with attention to key dimensions relevant to TB program implementation. These included:

- AI modality and intended clinical application,
- study design and population characteristics,
- reported diagnostic accuracy metrics,
- implementation outcomes or operational considerations,
- economic or policy implications.

Given the heterogeneity of study designs, populations, and reported outcomes across AI applications, formal quantitative synthesis or meta-analysis was not attempted. Instead, evidence was synthesized qualitatively across thematic domains corresponding to different stages of the TB diagnostic pathway.

### 2.5. Assessment of Methodological Limitations

Recognizing the methodological variability in AI diagnostic studies, particular attention was given to potential sources of bias that may influence reported performance. These include spectrum bias arising from case–control study designs, use of vendor-curated datasets, differences in imaging acquisition protocols, and potential dataset shift between training and real-world deployment environments [15,16,17].

Independent validation studies, benchmarking initiatives, and implementation studies conducted in routine screening populations were therefore prioritized when interpreting evidence relevant to policy and programmatic deployment [16].

## 3. CAD-CXR for Tuberculosis Screening and Triage

### 3.1. Policy Landscape and Scope of WHO Recommendations

The WHO recommends the use of CAD software to interpret digital chest radiographs for TB screening and triage among individuals aged 15 years and older in populations where systematic screening is recommended [3,4]. CAD is not intended to function as a stand-alone diagnostic test. Instead, it is meant to be embedded within diagnostic algorithms that include confirmatory microbiological testing and appropriate clinical oversight [3].

WHO recommendations for CAD-CXR are conditional and based on low to moderate certainty of evidence. Guidance emphasizes that the intended use, target population, and position of CAD within national screening pathways should be explicitly defined [3,4]. Current recommendations do not extend to children, reflecting insufficient evidence regarding diagnostic accuracy and clinical impact in pediatric populations [3].

Current WHO recommendations do not extend to children, reflecting insufficient evidence regarding diagnostic accuracy and clinical impact in pediatric populations [3,18]. Pediatric TB presents unique diagnostic challenges because radiographic manifestations often differ from adult disease. Children frequently present with intrathoracic lymphadenopathy, subtle parenchymal abnormalities, or non-specific radiographic findings, which can be more difficult to detect than the cavitary disease commonly seen in adults [18,19].

Extrapolating adult-trained CAD models to pediatric populations therefore raises both technical and ethical concerns, including potential diagnostic bias and reduced sensitivity [4]. Dedicated pediatric datasets and age-specific algorithm training will likely be required before CAD-CXR can be recommended for routine TB screening in children [4,18].

Priority deployment settings include community-based active case finding, high-throughput outpatient departments, prisons, and other congregate or high-risk environments where screening coverage is constrained by human reader capacity [2,3].

### 3.2. Evidence Underpinning Policy Recommendations

#### 3.2.1. Diagnostic Performance in Screening Contexts

In TB screening and triage, diagnostic performance is best interpreted in terms of sensitivity and its downstream implications for case detection and time to treatment, rather than relying primarily on summary accuracy metrics such as the area under the receiver operating characteristic curve (AUC). Screening tools are designed to identify individuals who should proceed to confirmatory testing, and their practical value depends on how effectively they capture people with disease while managing the workload placed on health systems [3,4].

Evidence synthesized for WHO policy development indicates that contemporary CAD-CXR products can be configured to achieve sensitivity levels comparable to those of human readers when used for adult TB screening [3,5]. However, performance varies across products, software versions, populations, and imaging conditions, and no single operating point appears optimal across all settings [3].

#### 3.2.2. Threshold Calibration as a Programmatic Decision

**Figure 1:**
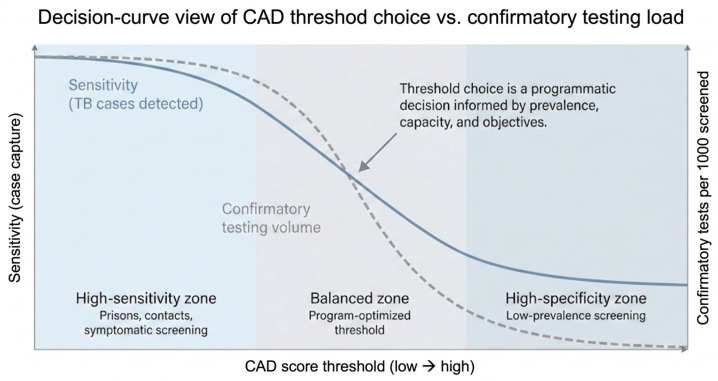
Decision-curve view of CAD threshold choice vs. confirmatory testing load. Computer-aided detection (CAD) systems for chest radiography allow programs to select an operating threshold that determines which individuals are referred for confirmatory tuberculosis testing. Lower thresholds prioritize sensitivity and maximize case capture but increase confirmatory testing volume, whereas higher thresholds reduce workload at the cost of missed cases. Optimal threshold selection is therefore a programmatic decision informed by local TB prevalence, screening objectives, and diagnostic capacity.

A defining feature of CAD-CXR systems is the ability to select an operating threshold that determines which images are classified as abnormal and referred for further testing. Threshold selection is therefore not simply a technical choice but a programmatic decision that reflects local priorities, TB prevalence, and available diagnostic capacity [4]. Thresholds that prioritize high sensitivity increase case capture but also raise confirmatory testing volume, whereas more specific thresholds reduce workload at the risk of missed cases [4]. The programmatic implications of these trade-offs are illustrated schematically in Figure 1.

WHO guidance recommends that programs define a sensitivity target appropriate to the screening context and calibrate CAD threshold locally using real-world data, rather than treating CAD output as a fixed or universally applicable score [4,13].

#### 3.2.3. Subgroup Performance and Sources of Variability

Diagnostic performance of CAD-CXR is not uniform across populations. Individuals with previous TB or chronic lung disease may generate higher false-positive scores. Conversely, people living with HIV and older adults may present with atypical radiographic features that affect sensitivity [3,20]. Device type, image acquisition protocols, and image quality further contribute to variability.

Disaggregated reporting and local verification are therefore essential to identify potential biases and mitigate inequities in access to diagnosis [2,3].

#### 3.2.4. Linking Diagnostic Performance to Patient- and Population-Level Outcomes

While much of the literature on AI for TB focuses on diagnostic accuracy, a smaller but growing body of evidence reports outcomes more directly relevant to patients and TB programs. Selected studies reporting patient- and population-level outcomes of AI-enabled TB interventions are summarized in Table 1, including evidence on screening throughput, confirmatory testing yield, cost per case detected [6,21,22].

Across studies, improved performance was associated with the use of large and diverse training datasets, integration of AI into structured screening workflows, and appropriate threshold calibration. In contrast, studies relying on curated datasets or lacking prospective implementation evaluation showed more limited generalizability and programmatic relevance.

Evidence linking AI deployment to programmatic response and patient-important outcomes (PIOs), including relapse-free cure, survival, health-related quality of life, and long-term functional recovery, remains limited. Pragmatic implementation studies and ongoing trials are increasingly prioritizing endpoints beyond diagnostic accuracy, including time to treatment initiation, treatment adherence, and downstream treatment outcomes [23].

Emerging evidence suggests that CAD-supported screening can improve program efficiency, but improvements in diagnostic performance alone are unlikely to translate into better patient outcomes unless AI tools are embedded within well-functioning diagnostic and referral pathways [3,7].

### 3.3. Validation, Benchmarking, and Local Verification

Independent validation is a critical safeguard for the safe and effective deployment of CAD-CXR. Performance estimates derived from enriched datasets or vendor-curated data may overstate accuracy relative to real-world screening population, where disease prevalence is lower and presentations are more heterogeneous [4,30].

Independent benchmarking platforms provide standardized mechanism for evaluating CAD products prior to procurement and scale-up. The FIND Validation Platform assesses CAD software using de-identified chest radiographs linked to clinical, microbiological, and expert reference standards derived predominantly from low- and middle-income countries [13]. Key features of independent and reproducible validation are summarized in Box 1.

Independent benchmarking does not replace the need for local verification. WHO guidance recommends short, prospective local validation studies using routine screening populations to confirm that selected thresholds achieve predefined sensitivity targets and to quantify resulting specificity and confirmatory testing volume [4,13]. Given that AI software evolves over time, explicit version control and re-verification following substantive updates are essential to maintain performance and patient safety [4].

Box 1 **FIND Validation Platform for AI-based diagnostic evaluation: why independent and reproducible evaluation matters. (https://www.finddx.org/what-we-do/projects/validation-platform-for-ai-based-diagnostic-evaluation/ (Accessed date: 7 February 2026)).**

- Independent evaluation avoids bias from vendor-developed datasets
- Uses real-world chest X-rays from multiple low- and middle-income countries
- Enables fair comparison of multiple CAD products under identical conditions
- Supports disaggregated performance analysis by key subgroups
- Provides reproducible evidence for policy, procurement, and version control

### 3.4. Risk of Bias and Dataset Limitations in AI Diagnostic Studies

Many AI diagnostic studies rely on curated or enriched datasets that may not reflect the clinical spectrum encountered in routine screening populations. Vendor developed datasets may contain higher proportions of radiographically obvious disease or images acquired under optimized imaging conditions, which can inflate reported diagnostic performance [16,31].

Spectrum bias and case–control study designs are common in early-stage AI research and may limit generalizability [15,31]. In addition, dataset shift changes in population characteristics, imaging devices, acquisition protocols, or disease prevalence can substantially alter algorithm performance after deployment [16,17].

Performance drift over time may also occur when software updates, imaging practices, or population characteristics change. These risks highlight the importance of independent benchmarking initiatives and local verification studies before large-scale programmatic use [16,17].

### 3.5. Economics and Procurement

For TB screening interventions, economic value is determined not only by diagnostic performance, but also by the ability to increase case detection and reduce delays to diagnosis at acceptable cost [2]. CAD-enabled chest radiography influences both referral patterns and resource utilization, making confirmatory testing volume a key cost driver [4].

Economic evaluations suggest that CAD-enabled screening may be cost-effective or cost-saving in certain contexts, particularly where asymptomatic TB is prevalent and screening throughput is high [21]. For example, cost-effectiveness analyses in India, Nigeria, South Africa and Zambia demonstrate substantial variation in cost per case detected depending on screening volume, diagnostic pricing, and health system capacity [6,21,24,25]. Cost-effectiveness estimates therefore vary substantially across countries depending on TB prevalence, labor costs, diagnostic pricing, and health system structure, and findings from one setting may not be directly transferable to another. Local adaptation is therefore essential for programmatic decision-making.

Procurement models for CAD software vary, including per-read licensing, per-device licensing, and bundled agreements. Programs should consider total cost of ownership, including hardware maintenance, software updates, training, and data governance, to ensure sustainability and value for money [2].

The economic impact of CAD-enabled screening depends on several cost components including imaging hardware, software licensing, confirmatory diagnostic testing, human resources, and programmatic overhead. These costs may vary depending on screening volume, procurement arrangements, and integration with existing TB diagnostic pathways [21,32].

Threshold calibration also influences costs because lower CAD score thresholds increase sensitivity but generate higher referral volumes for confirmatory testing such as Xpert MTB/RIF or culture [21,25]. Programs must therefore balance diagnostic sensitivity with downstream diagnostic capacity and budget constraints when selecting operational thresholds [25].

**Figure 2:**
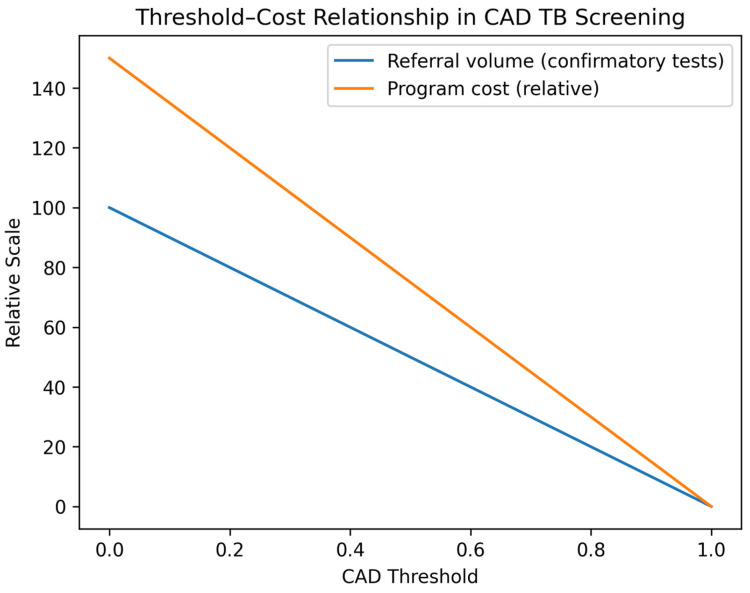
Relationship between CAD threshold selection, confirmatory testing volume, and downstream program costs in tuberculosis screening.

The relationship between CAD threshold selection, confirmatory testing volume, and downstream program costs is illustrated in Figure 2.

Lower CAD score thresholds increase sensitivity and case detection but substantially increase referral volume for confirmatory testing, leading to higher diagnostic costs and resource utilization. Higher thresholds reduce referral volume and costs but risk missed cases and delayed diagnosis. Optimal threshold selection therefore reflects a balance between diagnostic performance, available confirmatory testing capacity, and programmatic budget constraints.

### 3.6. Implementation Considerations

#### 3.6.1. Operational Considerations

**Figure 3:**
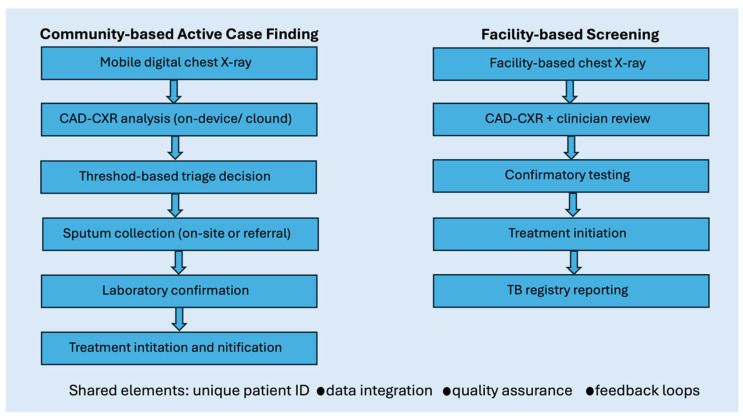
Pixels-to-patients workflow for CAD-enabled tuberculosis screening in active case finding and facility-based settings.

In TB care, the real-world impact of AI depends on both diagnostic performance and how well tools are integrated into routine health system workflows. CAD-CXR must be embedded within clearly defined diagnostic pathways linking screening to confirmatory testing, and treatment initiation [2,7]. The translation of AI outputs into clinical action across screening and facility-based workflows is illustrated in Figure 3.

The figure illustrates how computer-aided detection (CAD) for chest radiography can be integrated into end-to-end TB care pathways. In community-based active case finding and facility-based screening, CAD output informs triage decisions that trigger confirmatory testing, linkage to treatment, and reporting to national TB registries. Programmatic impact depends on effective integration of imaging, laboratory services, clinical oversight, and data systems rather than algorithm performance alone.

#### 3.6.2. Regulatory Considerations

AI software used for TB screening is typically regulated as Software as a Medical Device (SaMD). Regulatory requirements vary across jurisdictions but generally require demonstration of analytical validity, clinical validity, and ongoing post-market surveillance prior to clinical deployment. These processes are intended to ensure that AI systems meet established standards for safety, reliability, and clinical effectiveness [30,33].

Regulatory agencies increasingly require lifecycle monitoring of AI systems, including documentation of software updates, continuous performance monitoring, and mechanisms for reporting adverse events. Programs deploying AI tools for TB screening must therefore ensure compliance with national regulatory frameworks and international standards governing medical software, particularly when algorithms are updated or deployed across different populations and imaging environments [17,33].

#### 3.6.3. Equity Considerations

Equity considerations are central. Programs should monitor CAD performance by subgroup and adjust thresholds or workflows to mitigate unintended disparities [24,30]. Robust data governance, clear contractual arrangements, and continuous performance monitoring are essential to maintain trust and effectiveness over time. A practical governance checklist adapted for TB screening is provided in Box 2.

Box 2 **Governance checklist from WHO principles adapted to TB screening (World Health Organization).**

- Define intended use, target populations, and clinical role of AI
- Ensure human oversight and accountability for AI-informed decisions
- Use independently validated tools and conduct local verification
- Monitor performance across subgroups to promote equity
- Protect data privacy and define data ownership
- Plan for sustainability, version updates, and post-market surveillance

## 4. Beyond CXR: CT, Ultrasound, Cough Sound, and Digital Stethoscopes

### 4.1. AI-Assisted Computed Tomography

AI-assisted analysis of chest computed tomography (CT) images has been explored mainly for disease detection, severity assessment, and differential diagnosis in pulmonary tuberculosis. Several deep learning models have demonstrated the ability to detect active TB, quantify disease burden, and distinguish TB from other lung conditions such as malignancy or inactive TB disease. Reported correlations between AI-derived severity scores and radiologist assessments suggest that these tools have demonstrated potential in selected clinical situations, particularly where disease extent or treatment response requires more detailed characterization [11,34].

At the same time, the role of CT-based AI in routine TB screening or broader programmatic use remains limited. CT imaging is resource-intensive, involves higher radiation exposure than chest radiography, and is generally unavailable in peripheral or community-based settings where screening needs are greatest. Current evidence therefore supports CT-based AI primarily as a research-stage or referral-level diagnostic tool, with potential value in specific clinical contexts rather than as a scalable screening solution.

AI approaches applied to CT imaging include both classification and segmentation models. Classification models aim to distinguish active TB from other pulmonary conditions, while segmentation-based approaches can localize lesions and quantify disease burden [35,36]. While these approaches may improve diagnostic precision in complex cases, they often require large, well-annotated datasets and high computational resources [36]. In addition, variability in CT acquisition protocols and limited availability of standardized datasets across settings may constrain generalizability [37]. These factors, together with cost and infrastructure requirements, currently limit the role of CT-based AI to referral-level or research settings rather than large-scale screening programs [3].

### 4.2. Point-of-Care Ultrasound

Point-of-care ultrasound (POCUS) has attracted interest as a TB diagnostic aid because it is portable, relatively low-cost, and does not involve ionizing radiation. AI-assisted interpretation of lung ultrasound images has been proposed as a way to reduce operator dependence and variability, potentially enabling wider use by non-specialist providers [38,39].

However, the available evidence for POCUS in pulmonary TB remains limited. A systematic review of lung ultrasound for TB diagnosis reported highly heterogeneous sensitivity estimates, very limited specificity data, and substantial risk of bias across studies, reflecting variation in reference standards, operator expertise, and image acquisition protocols [9].

Existing studies vary substantially in methodology, including differences in operator expertise, acquisition protocols, and reference standards, limiting comparability and generalizability of reported findings [3,9,38].

At present, there is insufficient evidence to support routine programmatic use of POCUS, with or without AI, for TB screening or diagnosis [3]. Further progress will likely require standardized acquisition protocols, clearer interpretation criteria, and prospective evaluation within defined diagnostic pathways.

### 4.3. Cough Sound Analysis

AI-based analysis of cough sounds has emerged as a novel, non-invasive approach for TB triage and, potentially, disease monitoring. Recent work has explored deep learning approaches using curated cough audio datasets linked to clinical and microbiological reference testing, supported by wider use of mobile recording tools and improved data collection pipelines. Compared with earlier proof-of-concept studies, these efforts reflect a gradual shift toward more structured dataset development and more rigorous evaluation of model performance [12].

Across studies, reported performance varies widely depending on dataset composition, recording conditions, and model architecture, highlighting the need for standardized data collection and independent validation across diverse populations [12,26,40,41,42].

Despite this progress, reported diagnostic performance of cough-based AI models varies widely across studies, devices, and recording environments. Many evaluations rely on internal validation conducted by technology developers, which may increase the risk of optimistic performance estimates and limit confidence in reproducibility. In addition, a substantial proportion of studies use case–control designs, where the selection of both cases and controls may not reflect the spectrum of disease and non-disease presentations seen in routine screening populations, thereby limiting generalizability. While cough analysis may hold promise as a low-cost triage or monitoring tool, particularly in community-based contexts, current evidence supports its classification as a pilot-stage technology requiring further independent validation and prospective assessment within routine screening pathways.

### 4.4. Digital Stethoscope and Lung Sound Analysis

AI-assisted analysis of lung sounds captured using digital stethoscopes has also been investigated for TB detection. Experimental studies suggest that deep learning models can classify lung sounds with high accuracy under controlled conditions, potentially reducing the inter-observer variability inherent in traditional auscultation [20,43].

Current evidence is largely derived from small or controlled datasets, and broader validation across real-world clinical environments remains limited [43,44].

Translation of these findings into clinically meaningful decision support, however, remains challenging. Lung sound interpretation by AI systems is not yet sufficiently robust or interpretable for routine use, particularly in primary care settings where background noise, device variability, and mixed pathology are common [44]. At present, digital stethoscope-based AI for TB should be regarded as early research-stage, with no established role in screening or diagnosis.

### 4.5. AI-Enabled Data Analytic Tools for Tuberculosis Risk Stratification

AI-enabled analytic models are increasingly being explored to estimate an individual’s risk of developing active tuberculosis using routinely collected clinical, demographic, and laboratory data. Unlike imaging-based tools, these approaches operate at the level of patient-level data and may support decisions regarding screening intensity, confirmatory testing, or preventive therapy across different populations.

People living with HIV (PLHIV) represent one of the most extensively studied use cases for such models, given their substantially elevated TB risk even in settings with widespread antiretroviral therapy [45]. Conventional approaches to TB risk assessment in PLHIV, including symptom screening, tuberculin skin testing, and interferon-gamma release assays, have limited ability to predict incident disease and may be challenging to implement consistently at scale. In this context, cohort-based studies suggest that machine-learning models using routinely collected data may provide improved discrimination compared with standard screening tools, potentially enabling more targeted testing or preventive therapy [8].

However, PLHIV represent only one potential application. Similar risk stratification approaches could be extended to other high-risk groups, including individuals with prior TB, close contacts of infectious cases, or populations in congregate settings. Across use cases, most models have been developed and evaluated within specific datasets, and their transportability across settings, health systems, and epidemiological contexts remains uncertain.

Before routine implementation, AI-based risk models will likely require external validation, local calibration, and clearly defined clinical pathways that specify how risk scores inform action. Without these safeguards, analytic complexity may increase without corresponding gains in patient-important outcomes.

### 4.6. AI-Assisted Interpretation of Genomic Data on TB Drug-Resistance

Advances in whole-genome sequencing (WGS) have transformed the surveillance and management of drug-resistant TB, but interpretation of sequencing data remains analytically demanding and resource-intensive. AI and machine-learning approaches have been applied to support the identification of resistance-associated mutations, improve prediction of phenotypic resistance, and accelerate analysis pipelines for both clinical care and public health surveillance [46,47].

WHO’s 2023 catalog of mutations in Mycobacterium tuberculosis provides a standardized reference linking genetic variants to resistance phenotypes and underpins many current analytic tools. Building on this foundation, machine-learning models trained on large genomic datasets have shown improved accuracy in predicting resistance to first- and second-line TB drugs and in identifying rare or previously under-characterized resistance-associated mutations [10,29,48].

Despite these advances, AI-supported genomic interpretation should be viewed as decision support rather than a substitute for established laboratory and clinical expertise. Model performance depends on the quality and representativeness of training data, the completeness of mutation catalogs, and the transparency of analytic pipelines. However, differences in genomic datasets, sequencing platforms, and resistance prevalence across settings may influence model performance and require ongoing validation [46,47]. These considerations underscore the need for ongoing version control, auditability, and periodic re-evaluation of AI systems, particularly as resistance patterns and reference catalogues evolve [49].

## 5. Readiness for Policy and Programmatic Implementation of the Existing Modalities and Emerging AI Methodologies Relevant to TB Imaging and Diagnostics

### 5.1. Summary of Readiness and Programmatic Implications Across Modalities

The emerging AI technologies discussed above differ substantially in their level of policy endorsement, maturity of the supporting evidence base, availability of independent validation studies, and readiness for integration into routine TB programs. While CAD-enabled chest radiography has already been recommended by WHO and increasingly implemented by countries in the national TB screening programs, other AI modalities remain at earlier stages of research and validation. Table 2 summarizes the current policy status and programmatic readiness of major AI approaches relevant to tuberculosis diagnosis.

In contrast to CAD-enabled chest radiography, which is policy-endorsed and already in use at scale, AI applications involving CT, ultrasound, cough analysis, and digital stethoscopes remain at earlier and more heterogeneous stages of development. Although each modality addresses important gaps in TB care, the current evidence base does not support routine programmatic deployment. Clear differentiation between policy-ready tools and research-stage innovations is therefore essential to avoid misapplication and to guide responsible investment, evaluation, and future research.

Compared with imaging-based AI tools, risk models for PLHIV and AI-assisted genomic resistance interpretation address more focused, but clinically critical, decision points. Risk stratification tools may help prioritize preventive therapy and diagnostic testing among PLHIV, while genomic AI tools can enhance the speed and scale of drug-resistance surveillance and support regimen selection in specialized settings.

At present, both AI-based risk stratification models and AI-assisted genomic resistance interpretation tools should be regarded as emerging but increasingly mature applications. Although evidence supporting their technical performance and potential clinical utility is growing, broader programmatic adoption will depend on prospective evaluations demonstrating impact on individual patient outcomes (such as TB treatment success) or population-level outcomes (such as TB incidence and mortality), as well as successful integration into existing clinical, programmatic and laboratory workflows.

### 5.2. Emerging AI Methodologies Relevant to Tuberculosis Imaging and Diagnostics

Recent advances in artificial intelligence research have introduced several methodological developments that may enhance the reliability, interpretability, and clinical integration of AI systems used for tuberculosis (TB) detection. These developments include explainable artificial intelligence approaches, lesion segmentation models, data augmentation strategies, and automated radiology report generation systems. Although these techniques are not independent diagnostic tools, they play an important role in improving the performance and trustworthiness of AI-based systems used for TB screening and diagnosis.

In parallel with these methodological advances, a range of deep learning architectures have been applied to TB imaging tasks, each with distinct strengths, limitations, and roles within the diagnostic workflow. These architectures underpin many of the approaches discussed above, including classification, segmentation, and multimodal analysis. Table 3 provides an overview of commonly used AI architectures in TB imaging studies, highlighting their typical applications, strengths, and limitations.

Together, these methodological advances and architectural innovations are expected to improve the robustness, interpretability, and clinical integration of AI systems for tuberculosis screening and diagnosis.

#### 5.2.1. Explainable Artificial Intelligence and Model Interpretability

Deep learning models used for medical imaging often operate as complex “black-box” systems in which the internal reasoning behind predictions is not easily interpretable. Limited transparency may reduce clinician trust and hinder adoption of AI tools in clinical practice.

To address this challenge, explainable artificial intelligence (XAI) methods have been developed to provide insight into model decision-making processes. One of the most widely used techniques is Gradient-weighted Class Activation Mapping (Grad-CAM), which generates visual heatmaps highlighting image regions that contribute most strongly to the model’s prediction [50,51]. In TB detection models, Grad-CAM visualizations have been used to demonstrate that deep learning algorithms focus on radiographic features such as cavitation, consolidation, or nodular infiltrates that are clinically relevant to TB diagnosis [36,52,53,54].

Additional interpretability approaches such as SHAP (Shapley Additive Explanations) and LIME (Local Interpretable Model-Agnostic Explanations) have also been applied to medical AI systems to improve transparency and facilitate clinical validation [55,56]. While these techniques can help identify whether models rely on clinically meaningful features, interpretability tools remain imperfect and should be used alongside rigorous external validation and clinical oversight.

#### 5.2.2. Lesion Segmentation and Localization Models

In addition to image classification, deep learning methods have increasingly been applied to image segmentation tasks, which involve identifying and delineating specific anatomical structures or disease-related abnormalities within medical images. In TB imaging, segmentation models may be used to localize pulmonary lesions, quantify disease burden, and monitor treatment response.

The U-Net architecture, originally developed for biomedical image segmentation, has become one of the most widely used models for lung lesion segmentation [57]. Variants such as Attention U-Net and Mask R-CNN have been used to identify cavitary lesions, nodules, and areas of consolidation in chest radiographs and CT scans [35,36,58]. Segmentation outputs can be integrated with classification models to enhance diagnostic performance by improving lesion localization and feature representation [59].

Segmentation approaches may also facilitate quantitative assessment of disease severity by estimating lesion volume or lung involvement. However, these models typically require detailed pixel-level annotations for training, which are resource-intensive to generate and may limit the availability of large training datasets.

#### 5.2.3. Data Augmentation and Strategies to Improve Model Generalizability

A major challenge in AI-based TB detection research is the limited availability of large and diverse annotated datasets. To address this limitation, many studies employ data augmentation techniques, which artificially expand training datasets by applying transformations to existing images.

Common augmentation techniques include rotation, scaling, cropping, intensity adjustments, and noise injection, which introduce variability while preserving clinically relevant features [60,61]. More advanced methods use generative adversarial networks (GANs) to synthesize realistic medical images that can be used to augment training datasets [62].

Augmentation strategies have been shown to improve the robustness of deep learning models by exposing them to a wider range of imaging conditions during training [36,63]. However, inappropriate augmentation strategies may introduce unrealistic features or distort disease characteristics. Transparent reporting of augmentation methods is therefore essential to ensure reproducibility and accurate interpretation of AI performance.

#### 5.2.4. Automated Radiology Report Generation

Another emerging area of AI research involves automated generation of radiology reports from medical imaging data. Advances in multimodal deep learning and transformer-based language models have enabled systems that combine image feature extraction with natural language generation.

Early work on automated radiology reporting used convolutional neural networks combined with recurrent neural networks to generate structured reports [64]. More recent approaches incorporate transformer-based architectures, which have demonstrated improved performance in capturing complex relationships between image features and clinical descriptions [65,66].

In TB screening contexts, automated reporting tools could potentially assist clinicians by generating standardized descriptions of imaging findings and supporting documentation in settings where radiology expertise is limited. However, current systems may still produce inaccurate or incomplete reports and therefore should be considered decision-support tools rather than replacements for expert interpretation.

Together, these methodological advances are expected to improve the robustness, interpretability, and clinical integration of AI systems for tuberculosis screening and diagnosis.

## 6. Future Directions and Research Agenda

Despite substantial progress in the application of AI to TB screening and diagnosis, important evidence gaps remain. Addressing these gaps will be essential if AI-enabled tools are to contribute meaningfully to TB control while avoiding unintended harms or inefficient use of limited resources.

### 6.1. Expanding Evidence to Priority Populations

Current policy recommendations for CAD-enabled chest radiography are limited to individuals aged 15 years and older, reflecting persistent evidence gaps in children. Dedicated studies in pediatric populations are therefore a priority, with attention to age-specific radiographic patterns, disease presentation, and the suitability of available reference standards. Stronger evidence is also needed for adult subgroups in whom diagnostic performance and clinical impact may differ from general screening populations, including people living with HIV, individuals with previous TB, and older adults. In addition, future research should examine how CAD-enabled screening workflows manage non-TB abnormalities and whether AI tools can support appropriate referral pathways for differential diagnosis of other clinically important conditions detected on chest radiography.

Future studies would benefit from routine disaggregation of results by these subgroups and from evaluating whether tailored thresholds or complementary screening strategies improve both equity and effectiveness in specific contexts.

### 6.2. Moving Beyond Accuracy to Patient- and Population-Level Important Outcomes

Most published evaluations of AI-enabled TB tools continue to emphasize diagnostic accuracy, yet accuracy alone provides an incomplete picture of impact. Greater priority should be given to studies that assess patient- and population-level outcomes, such as time from screening to treatment initiation, treatment uptake coverage, impact on TB incidence and mortality, and cost effectiveness of interventions.

Pragmatic study designs, including cluster-randomized trials, stepped-wedge evaluations, and paired screen-positive studies, appear well suited to assessing these outcomes in real-world settings. Such approaches can capture how AI tools interact with health system capacity, diagnostic workflows, and patient behavior, generating evidence that is more directly relevant to policy and programmatic decision-making.

### 6.3. Strengthening Evidence for Emerging AI Modalities

AI applications beyond chest radiography, including point-of-care ultrasound, cough sound analysis, digital stethoscope data, and multimodal approaches, require more rigorous and standardized evaluation. Priority research needs for these modalities include the development of standardized acquisition and interpretation protocols, independent external validation using representative datasets, and prospective studies embedded within routine screening or care pathways.

Equally important is the transparent reporting of negative or neutral findings. Without this, there is a risk of premature adoption based on incomplete evidence, as well as inefficient allocation of research and development resources.

### 6.4. Governance, Safety, and Lifecycle Evaluation

As AI-enabled tools evolve through software updates and retraining, future research will need to address governance questions across the full product lifecycle. This includes methods for post-market surveillance, detection of performance drift, that is, unintended changes in diagnostic accuracy over time due to shifts in population characteristics, imaging practices, or software updates, assessment of subgroup bias over time, and evaluation of how humans and AI systems interact in clinical workflows.

Comparative studies of deployment models, such as cloud-based vs. edge-based processing, or centralized vs. decentralized governance, may also yield practical insights into trade-offs related to privacy, reliability, and sustainability across different implementation settings.

### 6.5. Integrating AI into Comprehensive TB Care

Looking ahead, the greatest potential value of AI may lie in its integration across multiple stages of the TB care cascade rather than in isolated applications. Multimodal models that combine imaging, clinical, laboratory, and programmatic data could support earlier detection, treatment initiation and monitoring, and post-TB care. At the same time, such approaches introduce additional challenges related to data interoperability, governance, and clinical accountability. Addressing these challenges alongside technical development is essential before consideration of broader adoption.

## 7. Conclusions

Artificial intelligence has evolved from experimental applications to increasingly practical tools within the tuberculosis diagnosis pathways, with CAD-enabled chest radiography representing the most mature and policy-endorsed use case. Evidence synthesized across a broad range of studies indicates that CAD-CXR can help standardize interpretation, improve screening efficiency, and facilitate earlier identification of individuals requiring confirmatory testing when integrated into well-functioning diagnostic pathways.

However, diagnostic accuracy alone is insufficient to determine real-world impact. The effectiveness of AI-enabled interventions depends on independent validation, local calibration, integration with clinical workflows, and alignment with available diagnostic capacity. Increasing evidence also highlights the importance of evaluating patient- and program-level outcomes, including time to treatment initiation, cost-effectiveness, and health system performance.

Beyond chest radiography, emerging AI applications, including CT imaging, ultrasound, acoustic analysis, risk stratification models, and genomic resistance prediction, demonstrate important potential but remain heterogeneous in evidence maturity and implementation readiness. Most of these AI-empowered applications require further independent validation and prospective evaluation before policy endorsement and routine programmatic adoption.

Taken together, current evidence supports a differentiated approach in which policy-endorsed tools such as CAD-CXR are scaled responsibly, while emerging technologies are advanced through rigorous evaluation frameworks. If implemented with appropriate governance, monitoring, and attention to equity, AI has the potential to contribute meaningfully to more timely, efficient, and person-centered TB diagnosis and care.

## Figures and Tables

**Table 1:** Studies reporting patient- and program-level outcomes of AI-enabled tuberculosis interventions.

Study	Setting and Population	AI Application	Study Design	Key Patient/Program-Level Outcomes	Main Findings
Moodley et al., 2022 [6]	Primary health clinics, South Africa	CAD-enabled digital CXR for TB screening	Prospective implementation study	Screening throughput; confirmatory testing yield	CAD-supported CXR improved screening efficiency and throughput in routine clinic settings, with acceptable referral volumes for confirmatory testing.
Velen et al., 2022 [22]	Correctional facilities, South Africa	CAD-enabled digital CXR	Prospective screening evaluation	TB yield; referral volume; operational feasibility	CAD in prisons identified additional TB cases beyond symptom screening and supported high-volume screening in a congregate setting.
Garg et al., 2025 [21]	Community-based active case finding, Nigeria	Ultraportable CXR with CAD	Economic evaluation alongside implementation	Cost per TB case detected; program costs	AI-enabled CXR screening was associated with lower cost per TB case detected than symptom-based screening in a setting with substantial asymptomatic TB burden.
Qin et al., 2019 [5]	Facility- and community-based screening, multiple countries	CAD-CXR	Comparative diagnostic study with operational implications	Inter-reader variability; workflow implications	CAD reduced inter-reader variability and achieved performance comparable to human readers, supporting use as a standardized triage aid in screening workflows.
Signorell et al., 2025 [23]	Community screening, Lesotho and South Africa	CAD alone vs. CAD + point-of-care CRP	Paired screen-positive pragmatic trial (protocol)	Time to treatment initiation; cost-effectiveness	Trial designed to evaluate downstream patient- and program-level outcomes beyond diagnostic accuracy; results pending.
Bartl et al., 2025 [8]	HIV care cohorts, sub-Saharan Africa	ML-based clinical TB risk model	Retrospective cohort analysis	Incident TB risk stratification	Model identified people with HIV at higher risk of incident TB than standard approaches, suggesting value for targeted testing or preventive therapy.
Kagujje et al., 2023 [24]	Adults with prior TB, Zambia	CAD-CXR for triage	Diagnostic accuracy study with subgroup analysis	False-positive referrals; subgroup performance	CAD performance differed among people with prior TB because of residual lung changes, with implications for threshold calibration and referral volume.
Raval et al., 2025 [25]	High-burden setting, India	AI-assisted chest X-ray interpretation (qXR, Genki)	Health technology assessment using retrospective screening data and literature-based diagnostic accuracy estimates	Cost per interpreted case; workflow efficiency; diagnostic accuracy	AI-assisted CXR interpretation showed favorable provider-side cost-effectiveness compared with conventional radiologist interpretation and may support programmatic triage where reader capacity is constrained.
Jaganath et al., 2025 (CODA TB DREAM Challenge) [26]	Outpatient clinics in 7 countries (India, Madagascar, Philippines, South Africa, Tanzania, Uganda, Vietnam)	Cough sound AI	Diagnostic challenge with independent external validation	Screening accuracy; reproducibility across datasets	The CODA TB DREAM Challenge showed that open, independently validated cough-based algorithms can be developed across multicountry data, strengthening the case for more rigorous external validation of acoustic triage tools.
WHO, 2025 [4]	High-burden screening settings	CAD products for chest radiography	Policy-informed evidence synthesis and multi-site comparative evaluation	Product variability; threshold selection; deployment considerations	Evidence shows substantial variability between CAD products and emphasizes the need for local threshold calibration and implementation-aware procurement decisions.
Kaewwilai et al., 2025 [27]	Clinical setting, retrospective TB and non-TB chest radiographs	AI-assisted CXR diagnosis and follow-up	Retrospective development/evaluation study	Differential diagnosis; follow-up utility; workflow support	Study suggests that AI-assisted CXR systems may support TB detection, differential diagnosis, and longitudinal comparison of imaging, with potential to improve workflow efficiency, although prospective implementation evidence remains limited.
Rajasekar et al., 2024 [12]	Curated cough datasets	Deep learning cough audio analysis	Model development and validation study	Triage potential; non-invasive screening feasibility	Cough-based deep learning showed promise as a non-invasive triage tool, but evidence remains dependent on curated datasets and needs prospective validation in routine pathways.
Wang et al., 2024 (TB-DROP) [28]	Large-scale whole-genome sequencing datasets of *Mycobacterium tuberculosis*	Deep learning for drug-resistance prediction	Genomic prediction study	Resistance prediction accuracy; laboratory decision support	Deep learning models demonstrated strong performance in genomic drug-resistance prediction and may support surveillance and regimen planning in specialized settings, but remain adjunctive to established laboratory and clinical workflows.
Pruthi et al., 2024 [29]	Large-scale TB whole-genome sequence datasets	ML/statistical resistance mutation characterization	Genomic analysis study	Resistance mutation identification; surveillance utility	Machine-learning approaches helped characterize resistance-associated mutations from large genomic datasets, supporting future AI-enabled surveillance and resistance interpretation pipelines.

**Table 2:** Comparison of AI modalities for tuberculosis screening and diagnosis.

AI Modality	Policy Endorsement	Evidence Maturity	Independent Validation	Programmatic Readiness
CAD-CXR	WHO recommended	High	Yes	High
CT-AI	No formal WHO recommendation	Moderate	Limited	Referral-level
Ultrasound AI	No formal recommendation	Low	Limited	Experimental
Cough AI	No formal recommendation	Low	Minimal	Pilot stage
Lung sound AI	No formal recommendation	Low	Minimal	Research stage
Genomic AI	Emerging guidance	Moderate	Increasing	Specialized laboratories

**Table 3:** Common artificial intelligence architectures used in tuberculosis imaging studies.

AI Architecture	Typical Application in TB Studies	Strengths	Limitations
Convolutional Neural Networks (CNN)	Classification of chest radiographs for TB detection	Efficient feature extraction; strong performance on imaging tasks	Limited interpretability; requires large annotated datasets
U-Net and variants	Image segmentation of TB-related lung abnormalities	Enables localization of lesions and improves explainability	Requires detailed pixel-level annotations
Attention U-Net	Segmentation with attention mechanisms focusing on relevant regions	Improved lesion localization	Higher computational complexity
Mask R-CNN	Object detection and lesion localization in radiographic or CT images	Accurate detection of multiple lesion types	Computationally intensive
Vision Transformers (ViT)	Advanced image classification and multimodal analysis	Captures global contextual relationships across images	Requires large datasets and computing resources

## Data Availability

No new data were created or analyzed in this study.
